# Cold-Cured Epoxy-Based Organic–Inorganic Hybrid Resins Containing Deep Eutectic Solvents

**DOI:** 10.3390/polym11010014

**Published:** 2018-12-22

**Authors:** Francesca Lionetto, Alessia Timo, Mariaenrica Frigione

**Affiliations:** 1 Department of Engineering for Innovation, University of Salento, via per Monteroni, 73100 Lecce, Italy; mariaenrica.frigione@unisalento.it; 2 Blue Think S.p.A., Corso Vinzaglio 12, 10121 Torino, Italy; timo.alessia@gmail.com

**Keywords:** cold-cured epoxy, deep eutectic solvents, ionic liquid, nano-composites, organic-inorganic hybrids, sol–gel technique, thermosetting resins, cross-linking

## Abstract

The development of improved cold-cured resins, to be used as either adhesives or matrices for FRP (fiber reinforced polymer) composites employed in the construction industry, has become the focus of several academic and industrial research projects. It is expected that the use of nano-structured organic–inorganic hybrid materials could represent a realistic alternative to commercial epoxy-based resins due to their superior properties, especially in terms of higher durability against: moisture, temperatures, harsh environments, and fire. In this context, organic–inorganic epoxy hybrids were synthesized by a modified sol–gel method without the addition of water. The experimental formulations were prepared starting from a mixture of a silane-functionalized epoxy resin, alkoxysilane components and a deep eutectic solvent (DES) based on a blend of choline chloride and urea. The latter was added in two different loads in order to analyze in depth its effect as a promoter for an effective dispersion of silica nano-phases, formed through hydrolysis and condensation reactions, into the cross-linked epoxy network. The produced formulations were cold-cured for different time spans in the presence of two hardeners, both suitable for a curing process at ambient temperature. In this first part of a wider experimental program, several analyses were carried out on the liquid (rheological and calorimetric) and cold-cured (calorimetric, thermogravimetric, dynamic-mechanical, flexural mechanical, and morphological) systems to evaluate and quantify the improvement in properties brought about by the presence of two different phases (organic and inorganic) in the same epoxy-based hybrid system.

## 1. Introduction

The use of epoxy resins as a matrix for composite materials or adhesives for structural bonding is widespread in the aeronautical and automotive industry. In these fields, the curing reaction of the thermosetting matrix takes place in controlled conditions (in terms of temperature, time, etc.) where monitoring is a topical issue [1,2,3]. Moreover, some factors during the manufacturing of structural bonded joints, such as surface treatments, curing cycle of the adhesive or entrapped moisture in the adherents, can strongly affect the long-term durability of bonded composite joints [4,5]. In the building industry, where the structures often placed outdoors are exposed to different conditions of temperature and humidity, the structural adhesive, generally used for the repairing of degraded concrete or to bond fiber-reinforced laminates to strengthen concrete components, must cure and harden in non-controlled conditions. Such adhesives are generally based on epoxy resins able to polymerize at room temperature (so-called "cold-cured") in the presence of suitable curing agents, i.e., aliphatic or cycloaliphatic amines [6]. As a consequence of the cold-cure process, the cold-curing epoxy resins present several disadvantages with respect to the typical heat-cured ones, such as: long periods of curing required to obtain satisfactory mechanical properties; incomplete polymerization; and a glass transition temperature (*T*_g_) of a few degrees (10–20 °C) higher than the common service (ambient) temperatures. Additionally, the *T*_g_ of the cold-cured epoxy resins may even decrease due to plasticization phenomena, i.e., when the resins are exposed to rain or to humid environmental conditions. The mentioned drawbacks make uncertain the durability of the cold-cured adhesives/matrices for fiber-reinforced polymer (FRP) composites, in particular when they are used in outdoor applications [7,8]. 

Therefore, the development of high performing/durable cold curing resins based on nano-structured epoxy resins represents a growing and attractive area in nano-engineered material science. Nanotechnology supplies opportunities to enhance material performance enabling the development of new polymeric materials with improved mechanical, thermal and functional properties, when compared to the pristine polymers [9,10,11].

Organic–inorganic (O-I) epoxy-based hybrids, obtained by intermingling at a nanometric scale an organic (epoxy) and an inorganic (mainly silica) components, has recently gained considerable attention as a means to produce high performing nano-materials. In the last years, the traditional approach employed for the production of epoxy-silica nano-composites, i.e., obtained from an epoxy where preformed silica particles are homogeneously dispersed, has been overcome by a technique based on the in-situ generation of siloxane domains by the sol–gel process, through the hydrolysis and condensation of metal alkoxides in aqueous solutions [12,13,14,15,16,17,18,19,20,21]. Since the epoxy-based O-I hybrids reported in literature polymerize at high temperatures, they are not suitable as "cold-cured" adhesives and matrices for FRP specifically intended for construction industry. 

On the other hand, the authors of the present study have recently developed O-I epoxy-silica hybrids, prepared by a non-aqueous sol–gel process, able to be cold-cured [22]. The modified non-aqueous sol–gel procedure entirely relies on the absorption of moisture from the atmosphere during operations of mixing, processing and curing in open air. The formation through the sol–gel process of inorganic co-continuous domains in epoxy–silica hybrids brought about an enhancement of the glass transition temperature and of the load-bearing properties of the obtained resins, even if cured at ambient temperatures [7].

In the last years, as reported by Donato et al. [23,24,25,26], the use of imidazolium-based ionic liquids for the synthesis of epoxy–silica hybrids with decreased random inter-particle aggregation and better morphology control, drove the interest in the use of ionic liquids in sol–gel technology [27]. Ionic liquids (ILs) are a large family of salts consisting of organic cations and anions of different nature, that melt at moderate temperatures, generally below 100°C [28,29,30]. Ionic liquids have emerged as versatile materials in several fields, due to their unique characteristics such as: low vapor pressure; good ionic conductivity; and thermal stability [30]. Several types of ionic liquids have been used as catalytic and curing agents for epoxy pre-polymers [31,32], in particular phosphonium-based ILs [33,34].

More recently, a new class of ionic liquids, known as Deep Eutectic Solvents (DES), has emerged. DESs are defined as a mixture of two or more components that, at a specific composition, are able to form an eutectic with a much lower melting point than those displayed by the individual components [35,36]. DESs share most of the properties of ionic liquids, such as negligible vapor pressure and wide electrochemical potential windows. In addition, DESs display some unique features, such as: ease of production; biocompatibility and biodegradability; low costs; and non-toxicity [37,38,39]. 

In this work, an eutectic mixture of choline chloride and urea has been used for the first time for the synthesis of organic–inorganic epoxy hybrids. This type of DES is easy to prepare and is biodegradable; in addition, the starting components are readily available and inexpensive [40]. Choline chloride (ChCl) is an organic salt based on quaternary amine, used as a vitamin supplement; it can be easily found with a relatively low cost [41]. Urea (U) is a common fertilizer as well as a product of the human metabolism [42]. The organic–inorganic hybrids were produced by a modified sol–gel method able to develop an in-situ formed nano-silica structure during the cross-linking reactions of a modified epoxy matrix taking place at room temperature. The sol–gel reaction, able to give rise to the inorganic nano-phase, occurred in the absence of water, employing ionic liquids based on deep eutectic solvents. Two kinds of epoxy-based O–I hybrids have been prepared employing two different hardeners, i.e., an aliphatic amine and a cycloaliphatic amine, in order to assess the ability of DES to promote the best dispersion of the nano-structured inorganic phase in the cold-cured epoxy matrix, without promoting its premature curing.

## 2. Materials and Methods 

### 2.1. Materials and Synthesis Procedures

**Figure 1:**
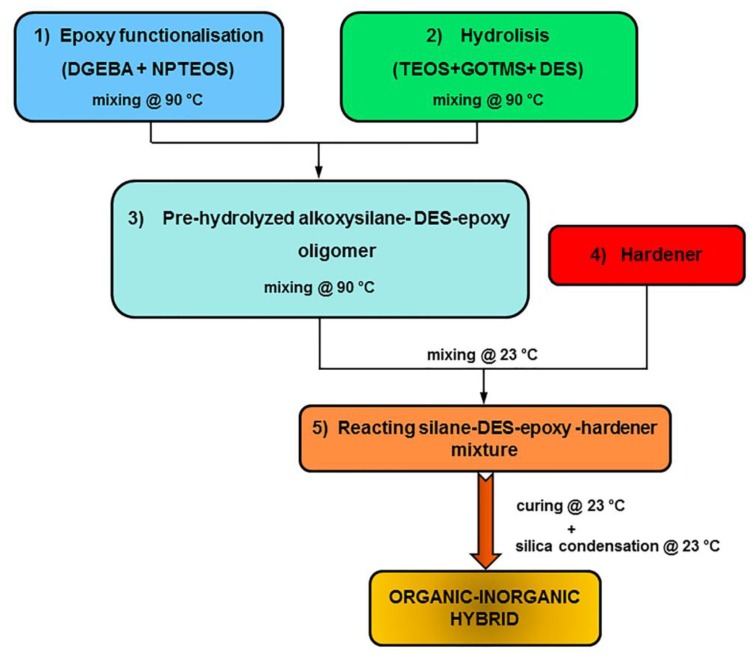
Steps of the synthesis process.

The procedure for the synthesis of the epoxy-based organic–inorganic hybrids consists of five steps, as schematized in Figure 1.

**Figure 2:**
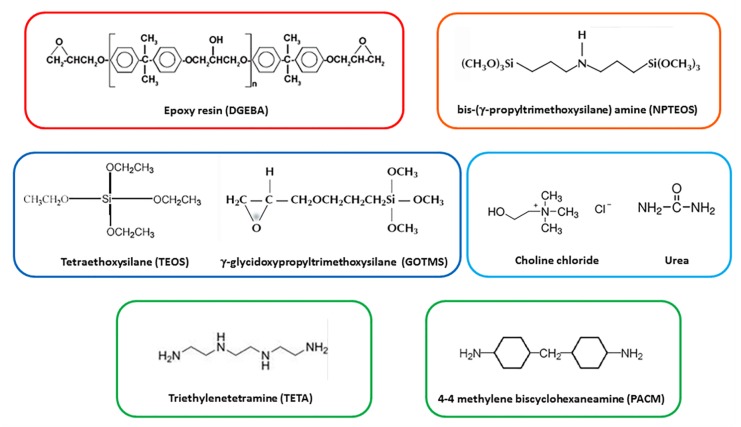
Chemical structure of the materials.

*1) Silane functionalization of the epoxy matrix*: a diglycidyl ether of bisphenol-A (DGEBA) with an epoxy equivalent weight of 184–190 g/mol was selected as an epoxy matrix; it is commercially known as Epikote 828 (Resolution Performance Products). The DGEBA was partially functionalized with a bis-(γ-propyltrimethoxysilane) amine (NPTEOS) obtained from Aldrich (Milan, Italy) (purity >90%) by stirring for 2 h at 90 °C. The chemical formula of each material used is reported in Figure 2.

*2) Pre-hydrolysis of a silane precursor-DES mixture*: the alkoxysilane precursors, required for the in-situ silica formation achieved by the sol–gel method, were based on a fixed combination (molar ratio of 1:0.12) of tetraethoxysilane (TEOS) and γ-glycidoxypropyltrimethoxysilane (GOTMS) obtained from Aldrich (purity >97%). In this step, the alkoxysilane precursors were mixed with a deep eutectic solvent (DES) based on a mixture of choline chloride (ChCl) and urea (U), both supplied by Iolitec GmbH (Heilbronn, Germany) (purity > 97%). The eutectic mixture was produced according to previous literature [28,29] by stirring at 90 °C a mixture of ChCl and U, with a molar ratio equal to 2:1, until a homogeneous clear colorless liquid was formed. This liquid mixture was, thus, able to remain liquid even at ambient temperature (freezing point: 12 °C). A mixture of TEOS and GOTMS with two different contents of ChCl-U was prepared at 90 °C under continuous stirring for 2 h. The selected DES contents were: 2.5 and 5 parts per hundred resins (phr), corresponding to 1.5% and 3% by weight of total mixture, respectively. It should be noted that steps 1 and 2 of the synthesis procedure were run at the same time in two different beakers.

*3) Pre-hydrolyzed alkoxysilane-epoxy oligomer mixture:* The two mixtures prepared in steps 1 and 2, i.e., silane-functionalized epoxy resin and TEOS/GOTMS/DES mixture, were mixed and stirred at 90°C for 2 h and then cooled to room temperature. The investigated formulations, whose compositions are summarized in Table 1, were labeled with L (lowest amount) and H (highest amount) according to the DES content, i.e., 2.5 and 5 phr, respectively. 

*4) Mixing of alkoxysilane-DES-epoxy mixture with the hardener*: A curing agent was, then, mixed to each silane-DES-epoxy mixture in order to induce the cross-linking reactions of the polymeric component of the mixture. To this aim, two different amine hardeners were selected, i.e., an aliphatic amine and a cycloaliphatic one. Triethylenetetramine (TETA), an aliphatic amine supplied by Elantas Italia S.r.l. (Collecchio, Italy) with the trade name IG 824-K24, and 4-4’ methylene bis-cyclohexanamine (PACM), a cycloaliphatic amine supplied by Aldrich, are both suitable curing agents for cold-curing of the epoxy resin. Each hardener was, then, added to the mixtures at ambient temperature, in order to avoid premature heat-curing, continuously mixing the liquid formulations for 30 min. The analyzed mixtures were labeled with a final “A” or “B” depending on the hardener used, TETA or PACM, respectively. 

For comparison purposes, two epoxy controls were also prepared: A neat epoxy system cured with TETA and a neat epoxy system cured with PACM, named Epoxy-A and Epoxy-B, respectively. Two different epoxy/hardener molar ratios were used for A and B systems: For the systems cured with TETA, a stoichiometric molar ratio epoxy:amine = 1:1 was selected; while for the systems cured with PACM, a molar ratio epoxy:amine =1:0.75 was chosen. The last selected value was, in fact, identified as the optimum ratio for a low temperature cure of coating systems [22,43].

*5) Matrix curing and silica condensation*: The final step in the synthesis of the organic–inorganic hybrids was the simultaneous cross-linking of the epoxy-based (organic) component of the mixture and the condensation of the siloxane (inorganic) domains for the in-situ production of silica. These two processes occurred both at room temperature. 

### 2.2. Production of the Specimens, Curing/Aging Procedures

All the fresh (i.e., liquid) mixtures, prepared as previously described, were immediately subjected to several characterization tests, as detailed in the next paragraph.

A part of the liquid mixtures was then deposited on a glassy substrate in the form of thin films (thickness around 0.8 mm) and cured/aged for prolonged times (up to one year) in laboratory-controlled conditions (i.e., 23 °C ± 2 °C and 55% ± 5% R.H.). These cold-cured specimens were employed for thermal analyses.

The remaining part of liquid mixtures was then poured into two different polytetrafluoroethylene (PTFE) molds, in order to produce rectangular specimens suitable for mechanical flexural tests (100 × 10 × 4 mm^3^) and for dynamic-mechanical tests (40 × 10 × 1 mm^3^). The specimens were cured for 14 days in laboratory conditions and subsequently aged in the same controlled environment (23 ± 2°C and 55% ± 5% R.H.) up to 4 months. 

### 2.3. Characterization of Fresh Mixtures and Cured/aged Specimens

A Differential Scanning Calorimeter (DSC, 822 Mettler Toledo, Columbus, OH, USA) was used to monitor the cure advancement and the evolution of *T*_g_ of the different systems, after different time spans, up to around 12 months. To this aim, the DSC experiments were carried out on fresh mixtures as well as on small samples taken periodically from the 0.8-mm cold-cured films. Thermal scans from 0 to 300 °C at a heating rate of 10 °C/min under nitrogen atmosphere (flow rate: 60 ml/min) were run on three specimens, at least, for each formulation produced, and the results were averaged.

The evolution of the viscosity of the uncured formulations at 40 °C was monitored over the time, in order to assess their gelation point. The test temperature of 40 °C was chosen to reduce the measuring time, which was several h at room temperature. A strain-controlled rheometer (Ares, Rheometric Scientific, Piscataway, NJ, USA) was employed for this purpose, performing the rheological tests at a shear rate of 0.5 s^-1^ with a parallel plate geometry (radius of the plate: 12.5 mm). Three runs were performed on each formulation. The value of the nominal ‘gel-time’ was taken as the time required by each mixture to achieve a viscosity equal to 1000 Pa·s [44,45]. 

Thermogravimetric analysis (TGA/DSC1 Stare System, Mettler Toledo) was also performed on cured specimens. The tests were performed from 30 to 800 °C, at a heating rate of 10 °C/min (sample weight about 15 mg). The experiments, at least three for each composition, were performed in air atmosphere on samples taken from films aged at room temperature for 4 months, in order to assess the decomposition temperatures for each system. The latter, in fact, can constitute a useful indication of the fire behavior of the resins. The results of thermogravimetric analyses were also employed to calculate the amount of silica residue in each mixture.

Dynamic-mechanical thermal analysis (DMTA) was carried out on the different samples cold-cured and aged for 4 months at ambient temperature. An ARES rheometer (Rheometric Scientific) was used to this aim, employing the torsion configuration. Samples of all systems (40 × 10 × 1 mm^3^) were subjected to an oscillation in torsion mode at a constant amplitude (1%) and frequency (1 Hz), by heating the specimens from 30 to 150 °C at a constant rate of 2 °C/min.

Flexural tests were carried out in a three-point bending mode, at a cross-head speed of 1.7 mm/min, with a span/thickness ratio of 16:1, using rectangular specimens (100 × 10 × 4 mm^3^) aged at room temperature for 4 months. The flexural tests were performed with a LLOYD LR5K machine according to the ASTM D790 standard [46]. At least five specimens were tested for each composition. The flexural strength (σ_fl_) and modulus (*E*_fl_) were calculated from the following equations:

(1) $$\sigma_{fl}=\frac{3*F_{\max}*L}{2*b*h^{2}}$$

(2) $$E_{fl}=\frac{F*L^{3}}{4*b*h^{3}*\delta }$$

where: *F*_max_ (N) was the load at failure, *L* (mm) the span length, *F* (N) and δ (mm) were the actual load and its displacement below the elasticity limit, respectively; *b* (mm) and *h* (mm) were the width and the height of the tested specimen, respectively.

Wide-angle X-ray diffraction of the powder, obtained from hybrid systems burned out in an oven at 500 °C in air atmosphere for 15 h, was collected on a PW 1729 Philips, Amsterdam, The Netherlands, using Cu Kα radiation in reflection mode (λ = 0.154 nm). The samples were step-scanned at room temperature from 2θ values of 10 °–50 °.

Finally, the morphology of the cured samples, fractured during the flexural tests, was examined by Scanning Electron Microscopy (SEM). A Zeiss EVO 40 Instrument (Zeiss, Oberkochen, Germany)was used at variable pressure and a voltage of 20 KV with the EDS system.

## 3. Results

### 3.1. Cure Kinetics of the Organic Component of the Hybrid Formulation 

**Figure 3:**
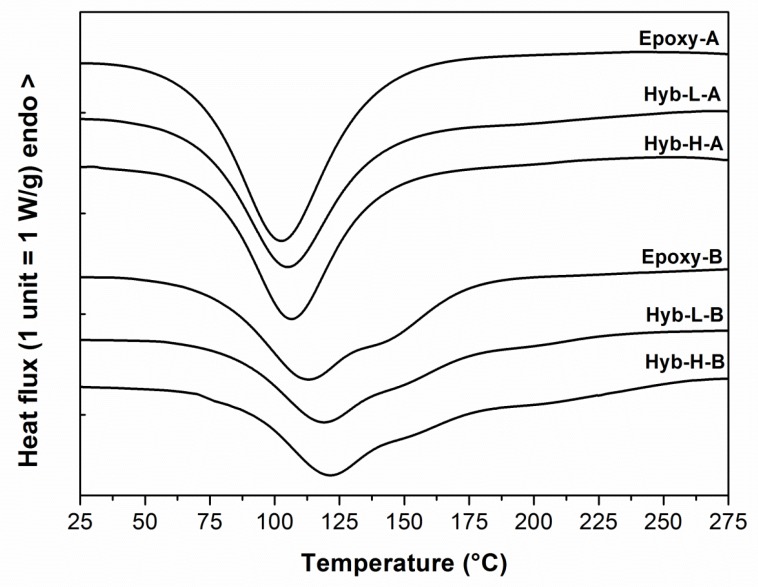
Dynamic DSC thermograms at 10°C/min of uncured epoxy systems as a function of DES content and curing agent typology.

The dynamic DSC thermograms of the two uncured epoxy controls and the relative hybrid systems, containing different DES contents and curing agents, are reported in Figure 3. The characteristic temperature and enthalpy values calculated by the calorimetric tests are summarized in Table 2. T_onset_ and T_peak_ reported in Table 2 are the onset exothermic temperature and the maximum exothermic temperature, respectively, obtained during the first heating scan performed at 10 °C/min. The enthalpy of curing (ΔH) is also reported. The glass transition temperature, T_g_, is obtained as the inflection point from a second DSC scan carried out at 10 °C/min.

As expected, the choice of aliphatic or cycloaliphatic amine has a significant effect on the curing kinetics of the epoxy-based systems. All the epoxy-based systems cured with the aliphatic amine, i.e., TETA, are characterized by an exothermic peak centered at 103–107 °C; the corresponding systems cured with the cycloaliphatic amine, i.e., PACM, exhibit a peak shifted toward higher values, centered between 113 and 122 °C. For the latter systems, furthermore, a shoulder between 140 and 175 °C is observed, which can be attributed to an increase of the reaction rate occurring at very high temperatures. This phenomenon was observed also by Van Assche et al. [47,48] for an epoxy resin cured in non-isothermal conditions in the presence of a cycloaliphatic amine (3,3’-dimethyl-4,4’-diaminodicyclohexylmethane). The ring structure of cycloaliphatic amine can be considered mainly responsible for the high values of *T*_onset_, *T*_peak_ and *T*_g_ observed for the B-epoxy systems cured with PACM hardener, which is more evident in the case of hybrid systems. Furthermore, both the control and hybrid systems cured with the aliphatic amine (TETA) exhibit higher values of enthalpy of curing compared to the corresponding systems cured with the cycloaliphatic amine (PACM). This difference is clearly evident for the control systems, where the ΔH of the Epoxy-A system is about 18% greater than that measured for Epoxy-B system; this difference is reduced (about 5%) in the case of the hybrid systems. All the investigated hybrid mixtures present a shift of *T*_onset_ and *T*_peak_ towards higher temperatures compared to the neat parent epoxy resins, due to the steric hindrance caused by silane-functionalized epoxy oligomers and the presence of alkoxysilane species and DES. 

The DES content is likely to affect the curing kinetics of the fresh reacting mixtures. As reported in Table 2, the values of Δ*H*, *T*_onset_ and *T*_peak_ slightly raise when the content of DES is increased from 1.5 % to 3%. This can be an indication of the occurrence of a reaction between the urea present in the deep eutectic solvent and the oxirane rings present in the epoxy oligomers and also in GOTMS coupling agent. Urea, in fact, can undergo thermal dissociation, generating corresponding amines and ammonium compounds that are able to react with epoxy groups and cause curing according to polyaddition and/or homopolymerization, respectively [35,49].

As mentioned, the glass transition temperature has been measured on each system completely cured in the first calorimetric scan through a second calorimetric experiment performed on the same specimen, again carried out at 10 °C/min. From the data in Table 2, it is possible to observe the effect on T_g_ of the hardener used to cure the epoxy and a further effect brought about by the hybridization in the presence of DES performed on each resin/hardener couple.

The significant reduction of the T_g_ measured only on the hybrid systems cured with TETA with respect to the control Epoxy-A (10–12 °C) could be ascribed to the plasticization of the network structure brought about by the presence of ChCl-U, with a consequent reduction in the effective cross-linking density. This result is coherent with what was previously observed in mixtures of neat epoxy-containing ChCl-U and cured with TETA, where a depression of T_g_ of 19 °C was found [35], and also with the results from experiments performed by other authors on epoxy blends containing other kinds of deep eutectic solvents [50,51]. 

In the hybrid formulations cured with PACM, the increase of the DES content from 1.5% to 3% leads to an increase in the maximum T_g_ which can be ascribed to a higher cross-linking density achieved, also thanks to the possible reaction between urea and the oxirane rings present in the epoxy oligomers and GOTMS coupling agent, as already underlined.

Referring to the measurements of gel-time obtained in rheological tests, the data reported in the last column of Table 2 show a general deceleration of the cross-linking reactions taking place in the hybrid systems with respect to what occurs in the neat corresponding epoxies, irrespective of the hardener used. The time required to achieve a viscosity of 1000 Pa·s in hybrid systems is, in fact, about 35% greater than the gelation time measured on the non-hybrid epoxy systems. This can be attributed to the fact that the sol–gel process, responsible for the in-situ production of the silica domains in the hybrid systems, occurs simultaneously with the curing reactions, thus retarding the progress of these latter. Moreover, the gel-time increases with the ChCl-U content. Finally, coherently with the exothermic curing peak observed in DSC curves, the gelation time is lower in the case of epoxy-based systems hardened with aliphatic amine TETA, while the epoxy-based systems hardened with a cycloaliphatic amine require higher temperatures and/or longer times with respect to epoxy-based systems hardened with an aliphatic curing agent.

**Figure 4:**
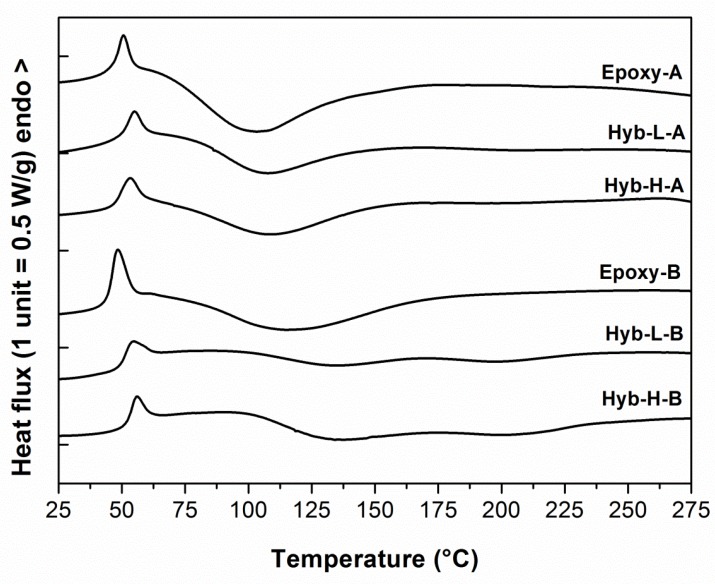
Dynamic DSC thermograms at 10 °C/min of hybrid and non-hybrid epoxy systems cured for 7 days at room temperature as a function of DES content and curing agent typology.

The dynamic DSC thermograms of the systems under analysis after a 7-day curing carried out at ambient temperature are reported in Figure 4. All the calorimetric curves display the typical behavior of cold-cured epoxy resins, featuring a first endothermic peak at the upper temperature side of the glass transition, manifested as a relief of the enthalpy acquired by the “physical aging” process during curing [52]. After the T_g_ interval, a broad exothermic peak at higher temperatures can be noticed. It is relative to the completion of the curing reactions, which are often not completed when the cure is performed at ambient temperature [53]. As observable in Figure 4, the glass transition temperatures measured for epoxy-based hybrid systems are higher than those measured on parent non-hybrid systems.

**Figure 5:**
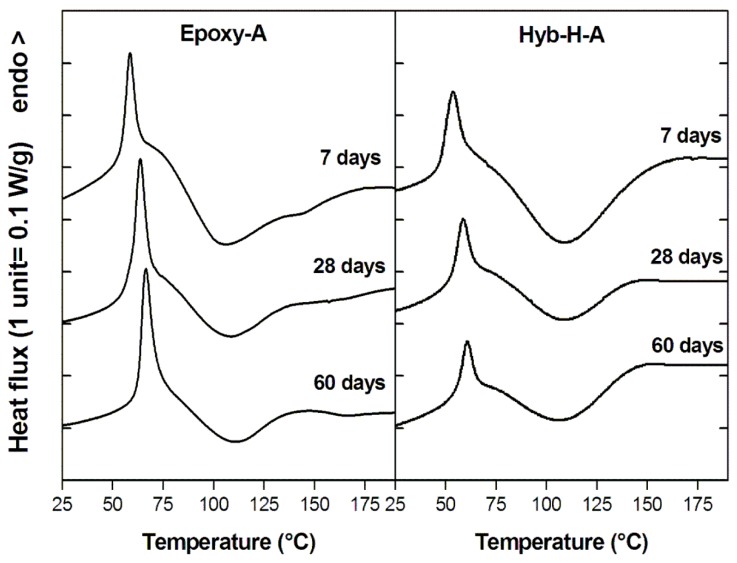
Dynamic DSC thermograms at 10 °C/min for Epoxy-A and Hyb-H-A systems aged at room temperature for different time spans.

It should also be noted that, irrespective to DES content and curing agent typology, the endothermic peak associated to the physical aging measured for an hybrid system is smaller compared to the same peak observed for the control resin, demonstrating that the evolution of the short range molecular order within the epoxide network resulting from physical aging has been hindered by the presence of alkoxysilane species. This is confirmed also by monitoring the aging at room temperature for longer times, as reported, as an example, in Figure 5 for the Hyb-H-A system in comparison with Epoxy-A.

**Figure 6:**
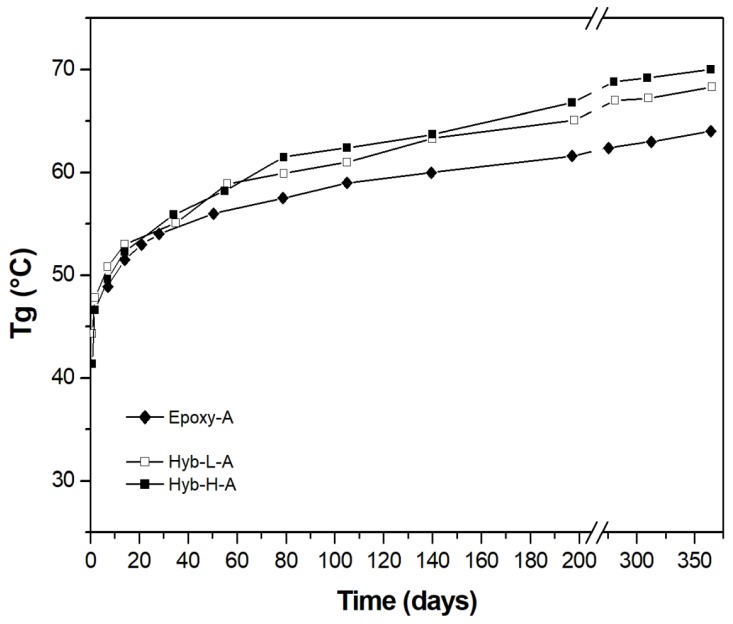
Temporal evolution of *T*_g_ as a function of DES content for epoxy systems cured with TETA hardener.

**Figure 7:**
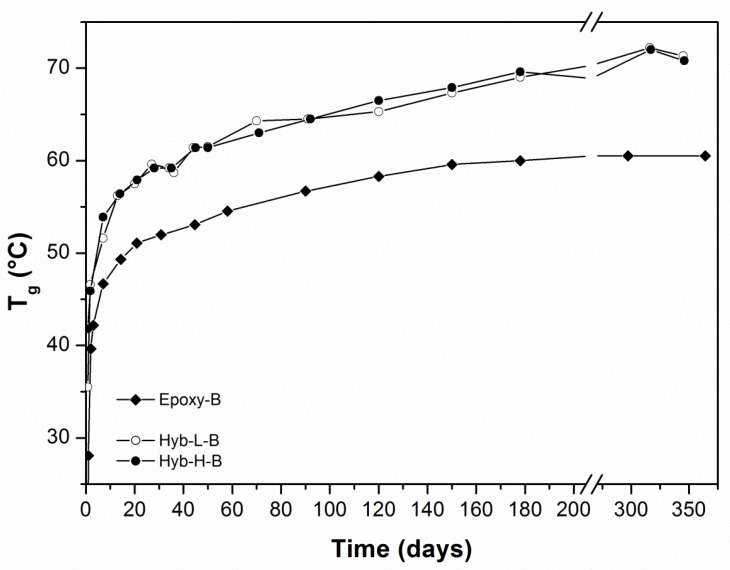
Temporal evolution of *T*_g_ as a function of DES content for epoxy systems cured with PACM hardener.

For practical applications, the evolution in time of the T_g_ for cold-curing resin systems is very important. Thus, in Figure 6 and Figure 7, the development of T_g_ during the aging taking place at ambient temperature is reported for the epoxy-based systems cured with aliphatic (Figure 6) and cycloaliphatic (Figure 7) amines, respectively, as a function of DES content. The T_g_ of the Epoxy-A system is initially lower than those measured for hybrid systems cured with the same hardener (TETA). This trend is maintained after aging at ambient temperature for about 1 year. The content of DES, finally, seems to have a small positive influence on the development of T_g_, this latter approaching 70 °C. After 1 year of curing, the curing process in the analyzed systems seems to not yet be stopped: the T_g_, in fact, is still increasing.

Passing to analyze the hybrid systems cured with PACM (Figure 7), both formulations are able to develop in shorter times T_g_ values much higher (even greater than 70 °C) than those measured for the relative non-hybrid epoxy system, irrespective to aging time and DES content. In this case, the process of cold-curing seems to be stopped after about 9 months, irrespective to the kind (non-hybrid or hybrid) nature of the system. As already mentioned in the Introduction section, the commercial cold-cured epoxy-based resins never achieve glass transition temperatures much higher than 60 °C, even after prolonged cold-curing times [54]. An increase in T_g_ of 10 °C represents an encouraging result proving the effectiveness of the presence of DES in a cold-curing epoxy resin.

### 3.2. Characterization of Organic-Inorganic Hybrid Samples

**Figure 8:**
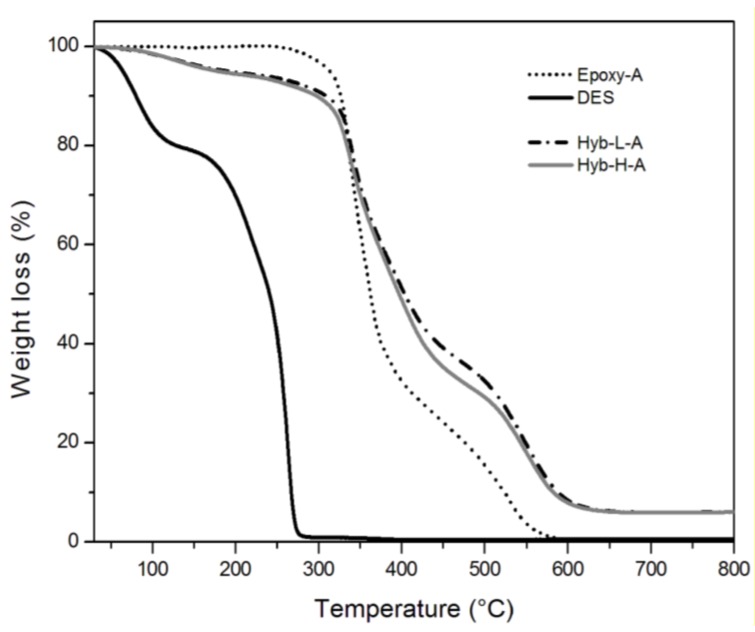
TGA thermograms of Epoxy-A and Hyb-H-A systems heated at 10 °C/min in oxidant atmosphere.

**Figure 9:**
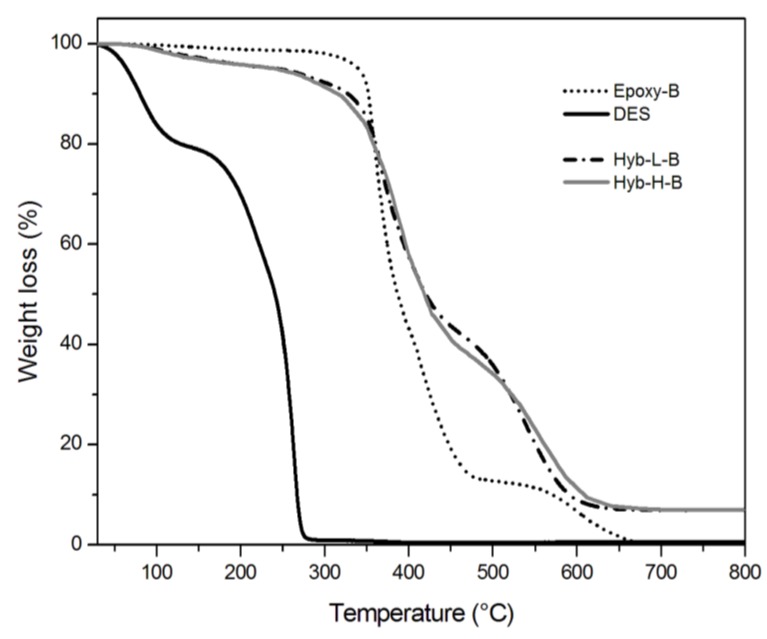
TGA thermograms of Epoxy-B and Hyb-H-B systems heated at 10 °C/min in oxidant atmosphere.

The behavior of the systems at very high temperatures, higher than the typical degradation temperatures, was, then, analyzed in the view to assess their thermal resistance. The thermograms relative to TGA analyses, performed in oxidant atmosphere on hybrid and control systems, are shown in Figure 8 and Figure 9 for the samples cured with TETA and PACM hardeners, respectively; some benchmark data are summarized in Table 3. The TGA thermogram of DES in isolation shows a first step of weight loss at about 100 °C, attributed to the evaporation of the water probably absorbed by choline chloride, which is very hygroscopic in nature. Then, a second sharper step of weight loss is observed in the 200–300 °C range, characterized by a first decomposition step at 210 °C and second one at 271 °C, attributed to the thermal decomposition of the ammonium cations (NH_4_^+^ at 235 °C) [55,56].

Referring to the hybrid systems, they undergo a first weight loss between 80 °C and 200 °C, irrespective to the DES content and the curing agent typology, differently from what is observed for both control systems, i.e., Epoxy-A and Epoxy-B. This loss is a measure of the amount of volatiles (water and mostly alkanols) that are given out through the condensation reactions within the siloxane domains. This phenomenon has been already reported in literature for other epoxy–silica hybrid systems obtained from the sol–gel process [12,22]. Furthermore, hybrid systems undergo a 10% weight loss at lower temperatures than those measured on the relative control systems; regardless to the kind of hardener, the difference between T0.1 values for the control and hybrid systems is about 20 °C for systems with the lowest content of DES and 30 °C for systems with the highest content of DES, respectively. This can be attributed to the thermal decomposition of ammonium cations associated with DES.

On the other hand, the hybrid systems exhibit a higher thermal resistance at very high temperatures with respect to control ones, as seen by the values of T0.5 reported in Table 3. The enhanced thermal stability was attributed to the presence of an inorganic siloxane structure, produced by the sol–gel method, which forms siliceous barrier layers able to inhibit, or at least limit, heat and mass transfer.

Referring to the effect of the curing agent, the hybrid systems containing ionic liquid cured with cycloaliphatic amine (i.e., Hyb-L-B and Hyb-H-B) show the highest thermal resistance; this could be attributed to the chemical stability of the hardener and to the formation of a more stable interconnected organic–inorganic microstructure. 

**Figure 10:**
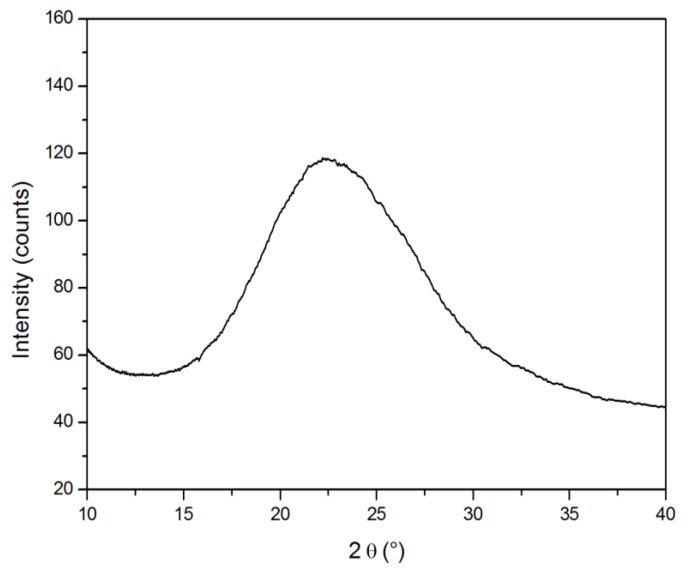
XRD pattern of Hyb-H-B obtained after its burn out at 500 °C.

The nature of the particles formed through the hydrolysis and condensation reactions, concurrent with the cross-linking of the organic component of the mixture, was also investigated. To this aim, samples of O-I hybrid systems were burned out at 500 °C for 15 h and then analyzed by X-ray diffraction (XRD) analysis. As an example, the XRD pattern of Hyb-H-B sample is reported in Figure 10. In this latter, a wide peak centered at 2θ = 22.5° is clearly visible, corresponding to the peak representative of amorphous silica, as reported in the literature [57]. 

**Figure 11:**
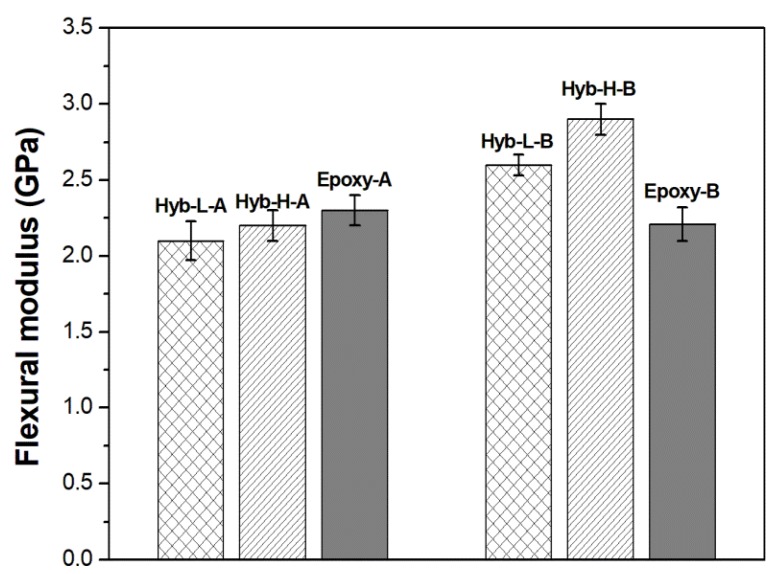
Flexural modulus of hybrid and non-hybrid epoxy systems.

**Figure 12:**
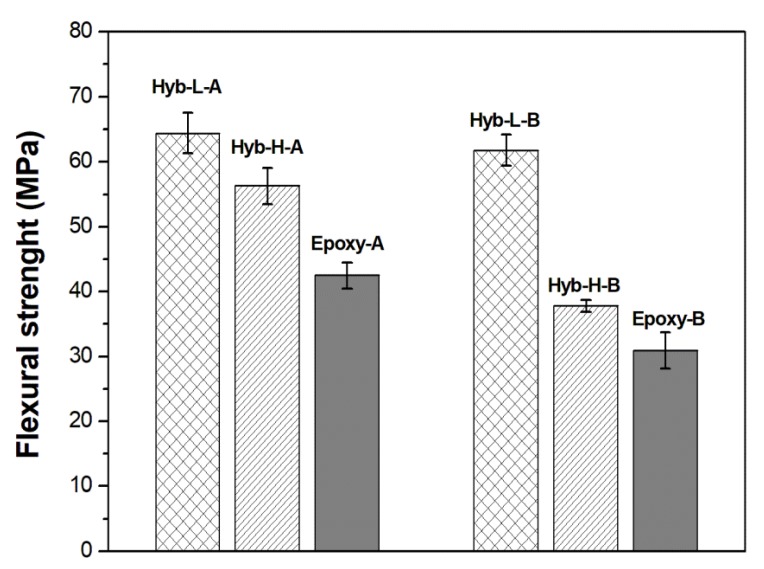
Flexural strength of hybrid and non-hybrid epoxy systems.

The comparison between the mechanical properties measured in flexural mode (i.e., modulus and strength) for the four hybrid systems and the respective non-hybrid resins, is shown in Figure 11 and Figure 12, respectively. The results of the flexural mechanical tests are summarized in Table 4 for all the analyzed systems.

Referring to the effect of the curing agent, the use of a cycloaliphatic amine allows to obtain cured epoxy with lower strength and strain in flexural mode, once again attributed to the ring structure of the cycloaliphatic hardener. The values of the modulus of the neat epoxy resin, cured with both curing agents, are in agreement with literature data [43,58]. 

The presence of a hybrid structure as well as the content of DES do not seem to influence appreciably the flexural modulus of the hybrid systems cured with TETA (Figure 11, Table 4). In the case of hybrid systems cured with cycloaliphatic amine, on the other hand, the flexural modulus is increased with respect to that measured on the parent resin with increasing the content of DES.

The positive effects in terms of flexural strength and strain linked to the introduction of ionic liquid in hybrid systems are more evident in systems with the lowest concentration of DES, regardless to the kind of hardener. The silica domains and the presence of a low amount of ionic liquid significantly increase the flexural strength in hybrid systems compared with the epoxy controls, as observed in Figure 12. 

**Figure 13:**
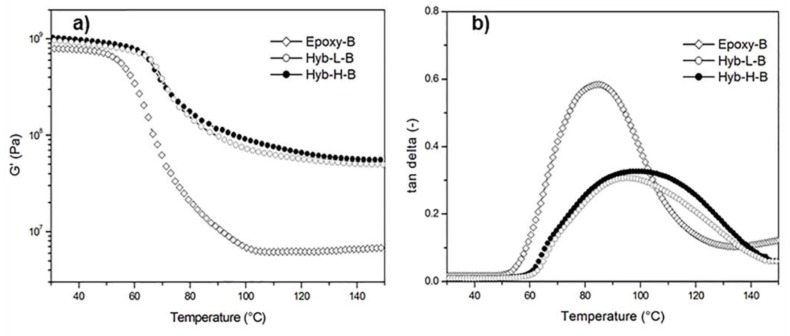
DMTA spectra: (a) Storage Modulus and b) tan delta of neat epoxy resin and of the O-I hybrids based on the same resins, all cured with the cycloaliphatic amine.

The results from dynamic–mechanical thermal analysis (DMTA), performed on the produced cold-cured hybrids, confirm the presence of a strong interconnection of organic and inorganic domains in the investigated materials. In Figure 13, the DMTA spectra (G’ and tanδ curves) of O-I hybrids cured with the cycloaliphatic amine are compared with the same curves obtained for the neat epoxy resin cured with the same hardener. The content of DES has a positive effect on the development of flexural modulus and shear modulus on the system cured with PACM hardener. The presence of siloxane domains in hybrid systems leads to a significant increase in the storage modulus G’, which is more evident at high temperature, i.e., in the rubbery plateau region. The tanδ curves of the hybrid systems show a significant increase in T_g_ along with a reduction of the peak height. These results indicate a hindering of the molecular motion of the organic chains brought about by the presence of nano-structured silica domains. The same results have been previously observed for other hybrid systems where the inorganic phase is produced by the sol–gel method using a coupling agent [20]. Moreover, in the current literature, it is reported that the use of the GOTMS as a coupling agent enhances the compatibility between the organic and inorganic phase by creating covalent bonds at the organic and inorganic interfaces, leading to silica domains homogeneously dispersed within the epoxy network as a co-continuous phase [14].

**Figure 14:**
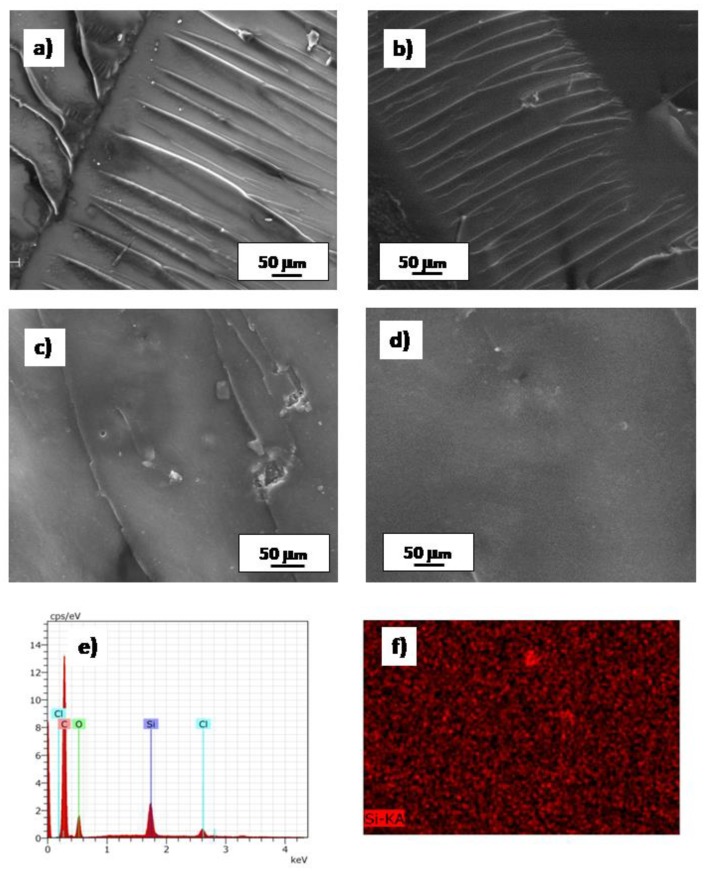
SEM images of: (**a**) Epoxy-A, (**b**) Epoxy-B, (**c**) Hyb-H-A and (**d**) Hyb-H-B; (**e**) EDS analysis of Hyb-H-B; (**f**) EDS map of the Silicon distribution in a Hyb-H-B sample.

The SEM micrographs of the fracture surfaces of neat epoxy and hybrid systems, broken during the flexural tests, are reported in Figure 14. The control epoxy resins, cured with TETA (Epoxy-A, Figure 14a) or with PACM (Epoxy-B, Figure 14b) hardeners, both exhibit the typical feature of brittle fracture behavior, due to the very limited fracture toughness characteristic of epoxy resins. The long cracks are mainly oriented in one direction, i.e., the direction of the applied load. Compared to the neat epoxy, the fracture surfaces of the hybrid systems show a significant different fractographic feature. As a representative example, the SEM micrographs of Hyb-H-A and Hyb-H-B are shown in Figure 14c,d, respectively. Figure 14d, in particular, displays the typical feature of organic–inorganic hybrids, consisting of diffuse silica nano-domains very well dispersed within an organic matrix, without any evident fracture line. Furthermore, this image also witnesses that the organic and inorganic phases are strictly interconnected, with no major macroscopic phase separation. Some microscopic and irregularly shaped agglomerations are also discernible. Similar SEM results were previously found for different epoxy–silica hybrids obtained by the same authors [22]. In that case, SAX analysis revealed the presence of nano-metric silica particles able to confer to hybrid polymer-enhanced flexural modulus and strength as well as transparency to visible light [22]. On the other hand, the SEM micrograph of the Hyb-H-A system (shown in Figure 14c) displays only a few crack lines and some irregularities, indicating the existence of some silica domains not yet homogenously dispersed in the organic matrix. 

Through EDS analysis, whose results are shown in Figure 14e,f, the homogenous presence of silica in the hybrid sample is confirmed, since the Si signal is homogenously displayed in each part of the analyzed sample. This latter observation also confirms the presence of co-continues nanostructured inorganic domains, perfectly interconnected in the organic matrix. The morphological analysis explains the differences observed in the mechanical properties of the hybrid samples cured with PACM although the inorganic residue content at 800°C is nearly the same. The different mechanical properties are therefore related not to a major content of inorganic silica domains but to a better dispersion and interconnection of these inorganic domains with the organic matrix, as a consequence of a different DES content. Deep eutectic solvents interact with the growing silica particles and epoxy network through hydrogen bonds. Differences in the size, geometry and Coulomb coupling forces between anions and cations are expected to affect the competing crosslinking and condensation reactions, contributing directly to the final silica particle size and morphology. The impact caused by deep eutectic solvent is still not fully understood and deserves further investigation

## 4. Conclusions

Organic*–*inorganic epoxy hybrids have been synthesized in view of producing cold-curing epoxy-based resins able to overcome some of the weaknesses characterizing the commercially available structural adhesives/matrices for fiber-reinforced composites, specifically intended for applications in construction. A modified sol*–*gel method has been proposed, which relies on a mixture of a silane-functionalized epoxy resin, alkoxysilane components and a deep eutectic solvent (DES), the latter based on a mixture of choline chloride and urea. The formulations have been cold-cured with two hardeners both suitable for the cure at ambient temperature, an aliphatic and a cycloaliphatic amine.

The different results obtained in the hybrid systems produced, hardened with aliphatic (A) or cycloaliphatic (B) amines, can be ascribed to the different cross-linking density and silica-based inorganic structure obtained in the two different systems, as a result of the balance of the kinetics of the two typical processes occurring in the system: Condensation reactions of the siloxane domains and cross-linking reactions of the epoxy oligomers. In the case of systems cured with aliphatic amine TETA, the action of DES seems to not be very effective in the formation of the O-I epoxy hybrid, probably because the cross-linking reaction is much faster than the condensation one, as demonstrated by the reduced gel-time measured for these hybrid systems (labeled with “A”) with respect to the others (“B” type). Furthermore, the addition of DES is likely to result in a plasticization of the network, with a reduction of *T*_g_ with respect to the Epoxy-A system; the elastic modulus, on the other hand, has results that are comparable to that measured on the non-hybrid resin. The weak balance between cross-linking reactions and siloxane condensation reactions is responsible also for the lower residual mass at 800 °C, indicative of a lower content of silica domains.

In contrast, in the case of systems cured with cycloaliphatic amine, i.e., PACM, a favorable balance between the kinetics of condensation and curing reactions is achieved due to a relatively slow cross-linking reaction; the inorganic phase, formed by the condensation process, is able to chemically bind to the growing epoxy network. In this case, the presence of DES in the systems cured with cycloaliphatic amine is beneficial for the dispersion of the in-situ generated silica, this being demonstrated by a general increase in *T*_g_, in the mechanical characteristics and in thermal resistance in the oxygen atmosphere. Although the role of dispersing agents of imidazolium-based ILs has been recently demonstrated for nanofilled epoxy systems [59,60], it is the first time that the beneficial effects of DES in the synthesis of O-I epoxy hybrids is reported.

The work presented in the manuscript is the first part of a wider experimental program aiming at developing cold-cured epoxy resins, to be used as either adhesives or matrices for FRPs employed in the construction industry. The analyzed O-I hybrid formulations’ results are very promising in the view of producing cold-curing epoxy-based resins able to overcome some of the well-known weaknesses characterizing the commercially available structural adhesives/matrices for fiber-reinforced composites specifically intended for civil engineering applications. Further studies will be devoted to a more systematic evaluation of the cold-curing kinetics and the final adhesive properties achieved by the experimented system after different curing times. Finally, the in-service adhesive performance of the hybrid resin if joined with other materials will also be investigated.

## Figures and Tables

**Table 1:** Compositions of the studied systems.

System	Ionic liquid content [phr]	Curing agent
**Epoxy-A**	0	aliphatic
**Hyb-L-A**	2.5	aliphatic
**Hyb-H-A**	5	aliphatic
**Epoxy-B**	0	cycloaliphatic
**Hyb- L-B**	2.5	cycloaliphatic
**Hyb- H-B**	5	cycloaliphatic

**Table 2:** DSC results for the neat epoxy resin and the hybrid epoxy-silica resin at different DES contents and curing agent typology.

System	Δ*H*^a^ (J/g)	*T*_onset_^a^ (°C)	*T*_peak_^a^ (°C)	*T*_g_^b^ (°C)	Gelation time at 40 °C^c^ (min)
**Epoxy-A**	510.1 ± 14.1	63.8 ± 1.0	103.2 ± 1.5	122.8 ± 2.5	48.0 ± 1.5
**Hyb-L-A**	461.9 ± 9.2	70.3 ± 2.3	105.4 ± 2.0	110.7 ± 2.3	63.2 ± 2.0
**Hyb-H-A**	493.9 ± 10.1	74.7 ± 1.9	107.2 ± 1.6	112.2 ± 1.9	67.4 ± 1.8

**Epoxy-B**	415.9 ± 8.4	75.7 ± 1.7	113.4 ± 2.0	126.5 ± 1.8	98.2 ± 2.3
**Hyb-L-B**	436.7 ± 7.3	78.5 ± 1.9	119.3 ± 1.8	145.8 ± 2.3	116.4 ± 2.6
**Hyb-H-B**	472.3 ± 9.1	85.4 ± 2.1	121.9 ± 1.7	152.4 ± 2.1	149.0 ± 2.7

^a^: Obtained from first DSC scan at 10 °C/min normalised with respect to the reactive sample weight (with the exception of TEOS content). ^b^: Obtained as an inflection point from the second DSC scan at 10 °C/min. ^c^: Obtained from chemorheology in correspondence of 1000 Pa*s.

**Table 3:** TGA results for the neat epoxy and the hybrid epoxy-silica resins at different DES contents and curing agent typology.

System	Residual weight at 800 °C (%)	*T*_0.1_^a^ (°C)	*T*_0.5_^b^ (°C)
**Epoxy-A**	0.1 ± 0	328.3 ± 1.5	362.6 ± 0.9
**Hyb-L-A**	5.1 ± 0.03	309.0 ± 1.0	403.7 ± 1.5
**Hyb-H-A**	5.3 ± 0.08	297.3 ± 1.0	397.2 ± 1.4

**Epoxy-B**	0.1 ± 0	351.7 ± 0.9	386.7 ± 1.1
**Hyb-L-B**	7.0 ± 0.07	327.3 ± 1.1	420.7 ± 1.5
**Hyb-H-B**	7.1 ± 0.08	316.0 ± 1.2	419.5 ± 1.6

**DES**	0.1 ± 0	80.2 ± 1.0	241.3 ± 1.0

^a^, Temperature in correspondence to 10% reduction of weight (heating rate: 10 °C/min). ^b^, Temperature in correspondence to 50% reduction of weight (heating rate: 10 °C/min).

**Table 4:** Mechanical results for the neat epoxy and the hybrid epoxy–silica resins at different DES contents and curing agent typology.

SYSTEM	FLEXURAL STRENGHT (MPa)	FLEXURAL STRAIN (%)	FLEXURAL MODULUS (GPa)
**Epoxy-A**	42.5 ± 2.0	1.9 ± 0.3	2.3 ± 0.1
**Hyb-L-A**	64.4 ± 4.1	3.7 ± 1.6	2.1 ± 0.9
**Hyb-H-A**	56.3 ± 3.2	2.7 ± 0.1	2.2 ± 0.1
			
**Epoxy-B**	30.9 ± 4.5	1.2 ± 0.1	2.2 ± 0.1
**Hyb-L-B**	61.8 ± 2.4	2.4 ± 0.1	2.6 ± 0.1
**Hyb-H-B**	37.8 ± 0.9	1.3 ± 0.2	2.9 ± 0.2
